# Macrophage peroxisomes at the crossroads of inflammation

**DOI:** 10.1097/IN9.0000000000000092

**Published:** 2026-10-20

**Authors:** Abhishek V. Thakkar, Irfan J. Lodhi, Edward B. Thorp

**Affiliations:** 1 Department of Pathology, Northwestern University, Feinberg School of Medicine, Chicago, IL, USA; 2 Division of Endocrinology, Metabolism and Lipid Research, Department of Medicine, Washington University School of Medicine, St. Louis, MO, USA; 3 Heart Center, Lurie Children’s Hospital, Chicago, IL, USA

**Keywords:** macrophage, peroxisome, inflammation

## Abstract

Macrophage plasticity of function enables the control of inflammation and its resolution. This plasticity is calibrated by the coordination of cellular signaling with intracellular metabolism. Peroxisomes are regulators of lipid metabolism, and emerging discoveries implicate these organelles in both the control of inflammation and macrophage polarization. Herein, we summarize the immunomodulatory functions of macrophage peroxisomes, with a particular focus on the integration of lipid metabolism. Highlighted are the diverse fates of inflammation due to peroxisomal activity and peroxisomal targeting as an emerging concept for regulation of inflammation and inflammatory disorders.

## 1. Introduction

Macrophages are professional phagocytes that internalize an array of extracellular material. This internalization requires macrophages to coordinate metabolism and detoxification of ingested material, with immune reactivity. Macrophage lipid metabolism is increasingly recognized as a critical regulator of inflammatory signaling, antimicrobial defense, and immune adaptation during infection. Peroxisomes are membrane-bound organelles that play central roles in lipid metabolism and reactive oxygen species (ROS) homeostasis, yet our understanding of their coordinated role in immune cells remains unclear.

Macrophages are among the first immune cells to respond to tissue injury and microbial infection, and their phagocytic capacity was described more than 140 years ago ^[1]^. Certain macrophages establish tissue residency during embryogenesis, where they help maintain tissue homeostasis ^[1]^. Other macrophage subsets are regularly replenished from the bone marrow and patrol the body to detect and respond to foreign threats ^[2]^. Macrophages play diverse roles during organ development ^[2]^, host defense during infection, and tissue repair after injury ^[3]^. During inflammation, they are activated via pattern recognition receptors and pathogen- or damage- associated molecular pattern receptors. This signaling activates pathways that include nicotinamide-adenine dinucleotide phosphate, reduced (NADPH) oxidase and NF-κβ (Nuclear factor kappa-light-chain-enhancer of activated B cells), leading to the release of cytokines and other biologically active mediators. These molecules orchestrate crosstalk with other immune cells and control the amplitude of the inflammatory response ^[4]^. Factors that regulate macrophage activation and transcriptional programming during inflammatory responses have been highlighted elsewhere ^[5]^. Importantly, cellular metabolism is tightly integrated with macrophage activation and polarization ^[6]^, highlighting the need to better understand how specific metabolic organelles and pathways regulate macrophage function during inflammation.

Peroxisomes are single membrane-bound organelles found in the eukaryotic cytosol and promote distinct functions and metabolic preferences. Peroxisomal proteins, called peroxins (Pex), are soluble cytosolic receptors that detect and bind to proteins that are then imported inside peroxisomal lumen. Pex5 imports proteins containing peroxisome-targeting sequence 1 ^[7]^ and Pex7 imports peroxisome-targeting sequence 2 proteins ^[8]^. However, these pathways also depend on each other for peroxisome import of proteins ^[9]^. Additionally, peroxisomes also incorporate catalytic oxidase enzymes within the lumen (see Table 1 for a list of the peroxisomal proteome) ^[10,11]^. The Pex proteins are vital for peroxisome biogenesis ^[12]^, import of matrix proteins ^[13]^, cellular redox homeostasis ^[14]^, and inter-organelle communication—which is necessary for metabolite transfer ^[15]^. The metabolic enzymes of peroxisomes play an active role in the breakdown and oxidation of fatty acids. Contrary to plants and fungi, where peroxisomes are the sole site for all fatty acid oxidation (FAO) ^[16]^, peroxisomes catabolize a specific subset of complex fatty acid substrates in human tissues ^[17]^. These substrates include very-long-chain fatty acids (VLCFAs) ^[18]^, branched-chain fatty acids (BCFAs) ^[19]^ and dicarboxylic acids ^[20]^. The peroxisomal FAO products are then modified ^[21]^ and transported to mitochondria for further metabolism ^[22]^. Such metabolic functions lead to the generation of ROS and reactive nitrogen species (RNS) as by-products. The by-products include superoxide (O^-^_2_), hydrogen peroxide (H_2_O_2_), and nitric oxide (NO) species ^[23]^. Such toxic moieties are then detoxified by peroxisomal ROS-quenching enzymes to maintain cellular redox balance ^[24]^. Contrary to their functions in lipid clearing, mammalian peroxisomes also harbor enzymes crucial for synthesis of ether lipids ^[25]^, bile acids ^[26]^, and steroids ^[27]^. The product of peroxisomal lipid biosynthesis form storage triglycerides and membrane phospholipids ^[28]^.

**Table 1.** Peroxisome proteome and function.

Function	Examples
Peroxisome membrane and biogenesis	PEX3, PEX12, PEX11a, PEX16, PEX2, PEX14, FIS1, PEX5, PEX6, PEX10, PEX11b, PEX1, PEX11g, PEX13, PEX5L, PEX26
Fatty acid and other metabolism	ABCD3, ACOT8, ACOX1, ACOX2, CAT, HACL, PHYH, DECR2, SCP2, PIPOX, CRAT, CROT, DAO, EPHX2, HSD17B4, ACOX3, ACSL1, EHHADH, GSTK1, AMACR, BAAT, HAO2, PECR, ABCD1, DHRS4, ECH1, NUDT12, IDH1, PRDX5, AGXT, ECI2, HAO1, ACOT4, HMGCL, DDO, ACAA1b, IDE, MLYCD, UOX, IDH2, NUDT19, ECI3, ACAA1a, PAOX, ACOT6, ACOT3, SOD1, ACSL5, ACOT1, FABP1, ABCD2
Biosynthesis	GNPAT, AGPS, HMGCL, FAR1
Other	PXMP4, ACBD5, LONP2, ACAD11, PXMP2, TMEM135, MARC2, ISOC1, SLC25A17, ALDH3A2, ZADH2, TYSND1, ATAD1, HSDL2, NUDT7, ACNAT1, MPV17L, SERHL, MPV17, GPD1, DHRS7B, MDH1, VWA8

Peroxisomes are found in all mammalian cell types, except red blood cells ^[29]^, and encompass a broad and diverse proteome. Their abundance is particularly high in metabolically active cells, including adipocytes ^[30]^, hepatocytes, and cells of the GI tract ^[31]^. The role of peroxisomes in highly metabolic tissues, such as adipose tissue, has been reviewed in detail ^[32]^. In humans, mutations in 14 of the 16 known Pex proteins cause peroxisome biogenesis disorders (PBD) that cause disease, highlighting their importance in normal body function ^[33]^. While peroxisomal metabolism is known to be important in shaping the response of highly metabolic tissues, the role of peroxisomes in the immune compartment is just beginning to be understood. Emerging evidence suggests that peroxisomes can exert both proinflammatory and inflammation-resolving functions in macrophages, depending on the cellular context and metabolic state.

The current literature on the role of peroxisomes in immune cells and distinct phases of inflammation also coincides with the development of state-of-the-art tools to study these organelles in vitro and in vivo. The conjugation of peroxisome-targeting sequence 1 (-SKL) with a combination of photosensitive and photoresistant green fluorescent protein (GFP) and red fluorescent protein (RFP), respectively, has allowed for the development of stable cell lines where turnover of these organelles by pexophagy can be causally studied ^[34]^. Such tools are very important, especially since pexophagy is responsible for 65% of peroxisomal biogenesis disorders ^[35]^. These tools have also now been used to study peroxisome turnover in vivo in the Drosophila model ^[36]^. More recently, these pexophagy sensors were utilized in inducible mouse models as well to show tissue-specific and temporal changes in pexophagy during development ^[37]^. To dissect the roles of more specific peroxisomal pathways, such as H_2_O_2_ production, genetically engineered proteins with photo-switching ability like Ro-GFP ^[38]^ and Hyper7 ^[39]^ have also been targeted to peroxisomes by conjugating PTS1 (-SKL) with their sequence. However, they have not been utilized for discovery of peroxisomal pathways in immune cells as of yet.

## 2. Proinflammatory roles of peroxisomes in macrophages

Peroxisomes were first found to be the site of localization of proteins like MAVS and antiviral signal transduction ^[40]^ in innate immune cells. Subsequently, peroxisomal Pex2 was found to be important for mast cell activation and release of inflammatory cytokines such as interleukin (IL)-6, tumor necrosis factor (TNF), and IL-13 ^[41]^. In myeloid cells, peroxisomes have been implicated in macrophage functions during inflammation, including phagocytosis ^[42]^. Macrophages express specialized receptors ^[43]^ that enable clearance of dead cells associated with injury or infection ^[44]^, whether derived from self or foreign sources. Recently, it has been shown that peroxisomes of professional phagocytes mediate cellular actin re-organization which supports cell mobilization and phagocytic cup formation during the inflammatory response to infection or injury ^[45]^.

Additional evidence implicates the mobilization of peroxisomes in macrophages during inflammation, potentially through mechanisms linked to lipid metabolism. In mammalian cells, peroxisomes are the sole site of metabolism of VLCFA, fatty acids with carbon chain lengths of 22 or more carbon atoms ^[46]^. Lipid metabolism by macrophages is an intricate component of the immune responses during infection ^[47]^ and tissue injury ^[48]^. Macrophages rely on VLCFA metabolism for inflammatory cytokine expression following activation ^[49]^ through an unknown mechanism. Interestingly, despite this defect in VLCFA metabolism and accumulation of fatty acids into triglycerides, the classically activated macrophages exhibited a higher number of peroxisomes than at the basal state ^[49]^. Such accumulation of peroxisomes may be explained by their importance in forming contacts with other organelles such as lipid droplets, mediating lipolysis, and production of inflammation-associated lipids (like prostaglandin E2) at later time-points following activation with lipopolysaccharide (LPS)/Interferron-γ (IFN-γ) or bacteria in macrophages ^[50]^. As such, the relationship between peroxisome numbers and their metabolic responses during inflammatory activation remains to be determined.

Peroxisomes have been shown to be key organelles for the synthesis of cell membrane phospholipids in multiple immune cell types, such as neutrophils ^[51]^, conventional dendritic cells, and B lymphocytes ^[52]^. Cells from patients with PBDs exhibit an imbalance in the ratio of fatty acids to cell membrane phospholipids ^[53]^. During the bacterial infection response, the peroxisome regulates the cell membrane lipid milieu, such as preventing the accumulation of phosphatidic acid from diacylglycerol. This supports the activation of a RhoA-like response, cortical cytoskeletal remodeling, and phagocytosis. Additionally, peroxisome control of RhoA signaling aids the release of inflammatory cytokines (such as IL-6, IL-1β, and TNF-α) from activated macrophages ^[54]^. The role of macrophage peroxisomes in cellular function has been initially described, as we highlight in Figure 1. However, many of these reported effects are due to the complete loss of peroxisomes during inflammatory activation of macrophages and do not shed light on how the individual metabolic functions of peroxisomes in macrophages might play a role in sustaining the inflammatory response.

**Figure 1.**
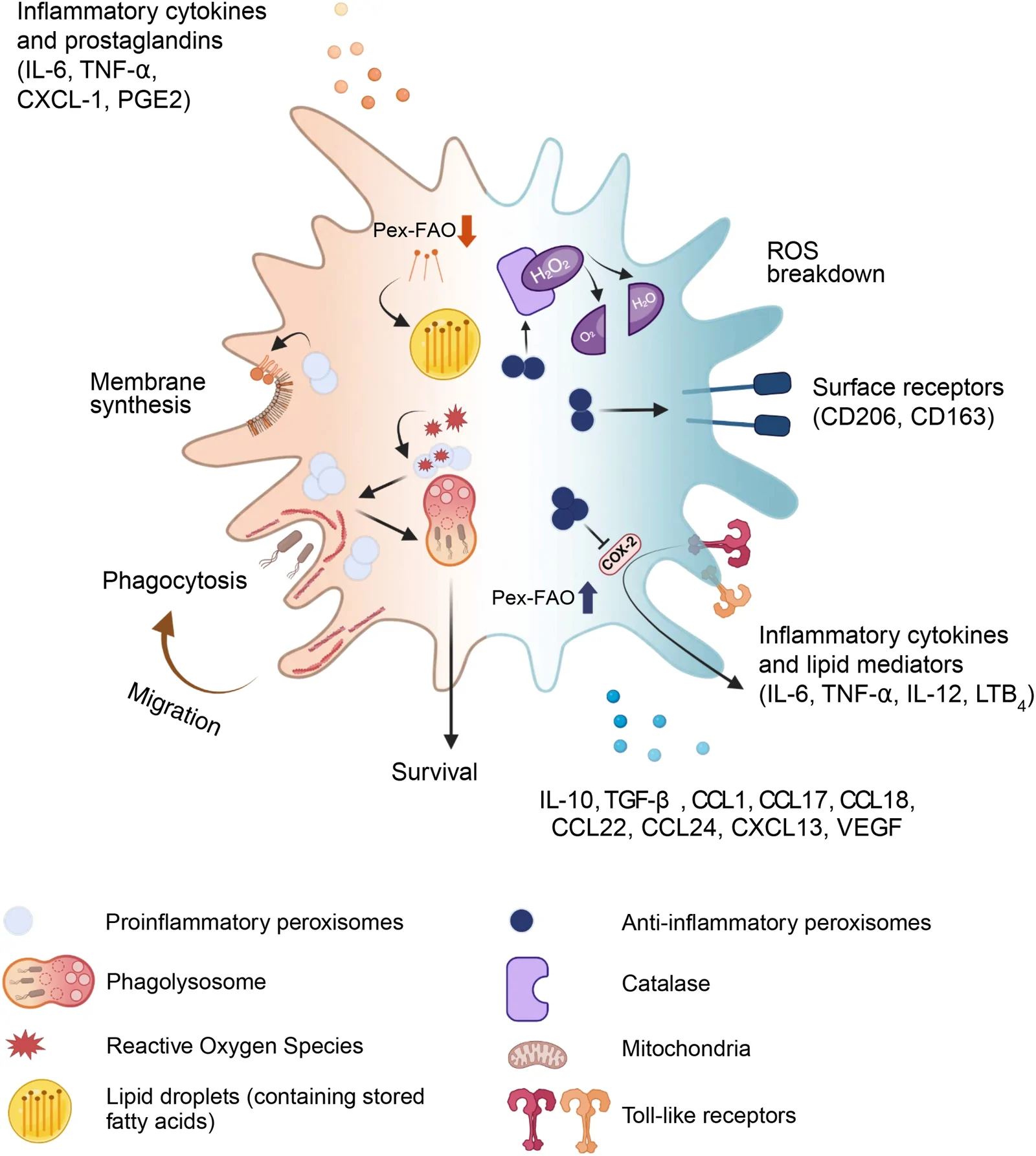
**Macrophage peroxisomes in inflammatory responses.** Peroxisomes aid in macrophage survival, migration, phagocytosis, phagolysosome maturation, cell membrane synthesis, and cytokine secretion during proinflammatory responses (left). Peroxisomes, however, also contribute to cytoplasmic ROS breakdown, maintaining mitochondrial health, regulating TLR-mediated responses, and anti-inflammatory cytokine secretion (right). IL, interleukin; ROS, reactive oxygen species; TLR, toll-like receptor.

It is likely that macrophage peroxisomes undergo remodeling during inflammation, and that these responses are shaped by external cues and tissue-specific inflammatory responses. Currently known macrophage peroxisomal metabolic pathways in inflammatory responses and the direction in which they influence inflammation are summarized in Figure 2. Further in-depth investigation of macrophage VLCFA and other fatty acid metabolic pathways in context of various in vivo stimuli is required to fully understand the role of the peroxisome in inflammatory response regulation. A broader characterization of the peroxisomal proteome in macrophages would also enable the illustration of context-specific macrophage peroxisome functions during inflammatory activation.

**Figure 2.**
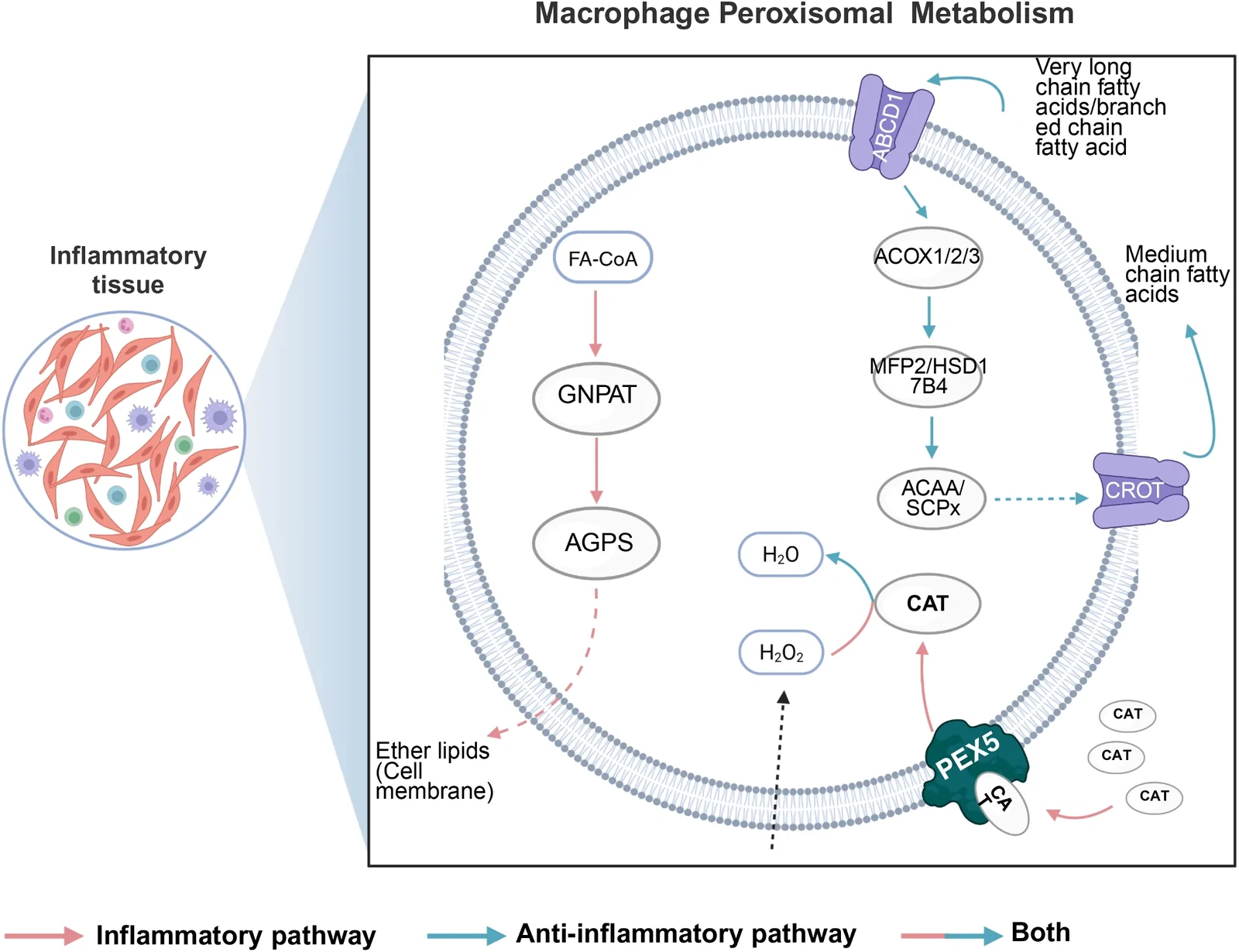
**Peroxisomal metabolic pathways in inflammatory responses.** A summary of metabolic pathways of peroxisomes showing pathways with only proinflammatory function (ether lipid synthesis), only anti-inflammatory functions (very-long chain fatty acid metabolism), and both functions (retention of catalase inside the organelle). ACAA, acetyl-CoA acyltransferase; ACOX, acyl-coenzyme A oxidase; AGPS, alkylglycerone phosphate synthase; CAT, catalase; CROT, carnitine *O*-octanoyltransferase; FA-CoA, fatty acid coenzyme A; GNPAT, glyceronephosphate *O*-acyltransferase; MFP2, multifunctional protein 2.

## 3. Roles of peroxisomes in inflammation resolution in macrophages

Peroxisome proliferator-activated receptors (PPARs) are a class of transcription factors that belong to the nuclear hormone superfamily and have a unique ability to bind intracellular lipid molecules and DNA for regulation of gene expression. These hypolipidemic molecules have emerged as important modulators of inflammatory responses ^[55]^, and their synthetic ligands, such as Clofibrate, have been shown to induce peroxisome proliferation ^[56]^. This could be due to the expression of PPARγ ^[57]^, PPARδ ^[58]^, and PPARα ^[59]^ by macrophages, their activation on ligand binding, and promoting transcription of peroxisomal genes containing PPAR response elements, such as *Acox1* ^[60]^. Such transactivation by PPARδ (in conjunction with liver-X receptors) was observed in response to lipids taken up from engulfment of apoptotic cells by macrophages (during efferocytosis), leading to prevention of autoimmune responses ^[61]^ and further clearance of dying cells ^[62]^. Additional PPAR-dependent molecular circuits, called trans-repression pathways, play a role in suppression of macrophage inflammatory functions. Unliganded PPARδ at baseline binds to and sequester an inhibitory transcription factor, B-cell lymphoma 6 (BCL6). Addition of PPARδ ligands or its genetic deletion frees up BCL6 for repression of inflammatory genes such as *Il1b, Ccl2, and Mmp9* ^[63]^. Similarly, PPARγ, on ligand binding and SUMOylation, interacts with corepressor complexes, nuclear receptor corepressor and SMRT (silencing mediated for retinoid and thyroid hormone receptor) to repress inflammatory genes even in the presence of proinflammatory stimuli ^[64]^. However, the functions of macrophage peroxisomes in initiation, progression, and amplification of such PPAR-mediated inflammation-modulating responses ^[65]^ are subject for further study.

The resolution of inflammatory cascades is dependent on quenching of ROS and reactive nitrogen species that are often generated during the acute stages of inflammation. Peroxisomal ROS-quenching enzymes include catalase (which contains a noncanonical peroxisome-targeting sequence) ^[66]^, superoxide dismutase 1 (which could be imported into peroxisomes with chaperone proteins) ^[67]^, glutathione S-transferase kappa ^[68]^, and low-molecular-weight antioxidants such as ascorbic acid ^[69]^ and plasmalogens ^[70]^. The noncanonical peroxisome-targeting sequence of catalase makes uptake of enzymes into peroxisomes inefficient during mild oxidative stress in cells, allowing cytoplasmic ROS to be detoxified ^[71]^. The peroxisomes of rat peritoneal macrophages, containing catalase, were first shown to surround a phagosome during phagocytosis of microbeads ^[42]^. More recently, this localization of peroxisomes surrounding a phagosome has been attributed functionally to clearance of oxidative burst generated following microbial infection ^[45]^. In neurodegenerative disorders and specifically within cerebral adrenoleukodystrophy (ALD), which is associated with reduced peroxisome biogenesis, uncontrolled inflammation results in blood-brain barrier disruption and white matter destruction ^[72]^. Studies using macrophage-like BV-2 microglial cells indicate that loss of peroxisome functions might contribute to dysregulated redox homeostasis, higher inflammatory activation, and secretion of cytotoxic factors that cause apoptosis in surrounding cell types during neural inflammation ^[73]^.

Another key feature of neurodegeneration associated with impaired peroxisome biogenesis is a marked reduction in VLCFA metabolism in monocytes and macrophages ^[74]^. Mutations in the ABCD1 gene, which encodes a peroxisome transporter for VLCFA uptake, predispose monocytes toward a proinflammatory activation, characterized by elevated TNF-α and IL1β transcription, higher tissue infiltration, and impaired induction of anti-inflammatory markers (CD206 and CD163) in differentiated macrophages following ingestion of myelin in X-linked ALD patients ^[75]^. Moreover, following inflammatory activation of macrophages in ALD, the uptake of free VLCFAs in the tissue caused activation of the JNK signaling pathway and release of matrix-degrading enzymes and chemokines. The ability of macrophages to clear the accumulation of VLCFAs during neuroinflammation by liver X receptor-mediated upregulation of ABCD1 is impaired, thereby prolonging the inflammation in ALD ^[76]^. Appropriate regulation of another rate-limiting enzyme in VLCFA metabolism, ACOX1, has also been found to be vital in proper functioning of glial cells and avoiding neuroinflammation. Patients with higher than optimum expression of ACOX1 had more ROS-mediated glial cell loss, and patients with ACOX1 loss-of-function mutations suffered from ALD likely through the aforementioned pathways ^[77]^. Hematopoietic stem cell transplantation, genetic treatment, and pharmacological interventions to restore the VLCFA metabolism have all been shown to be beneficial in restoring the anti-inflammatory functions of macrophages in ALD. However, such benefits exist only in macrophages cultured from ALD patients ex vivo ^[78]^ and to a limited extent in vivo ^[79]^.

The anti-inflammatory functions of macrophage peroxisomes also appear to be tissue-specific. Peroxisome proliferation during LPS stimulation reduces the COX-2 activation and subsequent proinflammatory cytokine secretion in macrophages derived from peritoneum, but not in those differentiated from bone marrow ^[80]^. Therefore, additional causal and in vivo studies linking peroxisomal fatty acid metabolism and macrophage anti-inflammatory functions are warranted. The currently known anti-inflammatory functions of macrophage peroxisomes and their associated metabolic pathways are summarized in Figures 1 and 2, respectively. Among the synthesis pathway of peroxisomes, the glyceronephosphate *O*-acyltransferase-mediated synthesis of plasmalogen C16-lysophosphatidylethanolamine (pLPE) and C16-alkanyl-lysophosphatidic acid (eLPA) in thymocyte peroxisomes has been shown to be important for selection and maturation of natural killer T (NKT) cells ^[81]^. However, whether pLPE and eLPA are also present in other organs and if their supplementation can enhance NKT cell activation, IL-10 secretion, and amelioration of inflammation remains to be seen ^[82]^.

## 4. Opportunities for intervention

Although substantial evidence demonstrates that peroxisomes and their metabolic functions influence multiple phases of inflammation, many questions regarding the regulation of peroxisome abundance and function remain unresolved. First, it is unclear whether peroxins that are responsible for the biogenesis of peroxisomes are regulated transcriptionally, post-transcriptionally or through post-translational mechanisms. Furthermore, it remains to be seen if there is heterogeneity in peroxisome composition within macrophages, and if so, whether it contributes to diverse immune responses in response to different stimuli. Characterization of the human macrophage peroxisomal proteome would further expand our current knowledge by identifying additional components of current peroxisomal pathways and facilitating the discovery of any uncharacterized immune functions of such pathways. Studying peroxisomal pathways in different macrophage subtypes, such as recruited and resident macrophages, could also be useful in unraveling the diversity of their contribution to immune responses and tissue-specificity by this organelle. Elucidating the crosstalk between peroxisomal biogenesis and metabolic pathways will advance our knowledge in understanding how these organelles coordinate with mitochondria and lipid droplets during immune responses. Investigating the organelle dynamics at a super-resolution level may provide critical insights into such complex mechanisms ^[83]^.

From a therapeutic perspective, vorinostat, a histone deacetylase inhibitor that induces ABCD2 expression and promotes uptake of VLCFAs in peroxisomes, has shown some promising results by reducing inflammatory cytokine IL12B secretion by macrophages and lowering CSF-serum ratios of immunoglobulin in cerebral X-linked ALD patients ^[78]^. A peroxisome proliferator drug, 4-PBA, has also been experimented for improving lung repair following SARS-CoV2 ^[84]^. There still remains a lack of nontoxic, cell-specific therapeutic strategies capable of selectively modulating peroxisomal responses to limit inflammation. Deep mechanistic studies of macrophage peroxisomes will also yield knowledge that can open therapeutic avenues in inflammatory disorders.

## 5. Conclusions

We have only started to understand the role of macrophage peroxisomes in how they sway inflammatory responses through studies in ALD and infections. Although causal studies linking macrophage peroxisomes and inflammation progression are lacking, there is currently evidence on both sides, that is, indicating a supportive role of peroxisomes in inflammation and in amelioration of inflammation. Beyond maintaining or mitigating inflammatory responses, macrophage peroxisomes can also mediate several aspects of inflammatory responses through their ability to interact with other organelles, control cellular redox homeostasis, and participate in cellular metabolic reactions. A characterization of the macrophage peroxisomal proteome at basal state and after activation would enable further identification of these organelle-specific pathways in inflammatory responses. Harnessing current state-of-the-art techniques to carry out more peroxisome-specific studies in vivo would also aid in mapping cellular peroxisomal circuits during inflammation. In conjunction with the above, macrophage peroxisomal pathway studies in human models and targeting those pathways are also on the horizon from a therapeutic perspective.

## Author contributions

A.V.T. and E.B.T. prepared the original draft of this manuscript. A.V.T., I.J.L. and E.B.T. reviewed and further edited the manuscript for submission. E.B.T. is the corresponding author on the manuscript.

## Conflicts of interest

The authors declare that they have no conflicts of interest.

## Funding

This review was supported by predoctoral fellowships 25PRE1195426 and F31HL177921 by the American Heart Association and National Heart, Lung and Blood Institute, respectively, to Abhishek Thakkar. The grants 24SFRNPCN1289611 and 1R35HL177401 by the American Heart Association and National Institutes of Health to Edward Thorp also supported this manuscript.
